# Effects of kiwi consumption on plasma lipids, fibrinogen and insulin resistance in the context of a normal diet

**DOI:** 10.1186/s12937-015-0086-0

**Published:** 2015-09-15

**Authors:** Jose I. Recio-Rodriguez, Manuel A. Gomez-Marcos, Maria C. Patino-Alonso, Elisa Puigdomenech, Blanca Notario-Pacheco, Nere Mendizabal-Gallastegui, Aventina de la Cal de la Fuente, Luis Otegui-Ilarduya, Jose A. Maderuelo-Fernandez, Angela de Cabo Laso, Cristina Agudo-Conde, Luis Garcia-Ortiz, on behalf of the EVIDENT Group

**Affiliations:** 1 La Alamedilla Health Centre, Castilla and León Health Service–SACYL, redIAPP, IBSAL, Salamanca, Spain; 2 Statistics Department, University of Salamanca, Salamanca, Spain; 3 Passeig de Sant Joan Health Center, Catalan Health Center, Barcelona, Spain; 4 Cuenca III Health Centre, Castilla La Mancha Health Service–SESCAM, Cuenca, Spain; 5 Primary Care Research Unit of Bizkaia, Basque Health Care Service-Osakidetza, Bilbao, Spain; 6 Casa del Barco Health Center, Castilla and León Health Service–SACYL, Valladolid, Spain; 7 Torre Ramona Health Center, Aragón Health Service–Salud, Zaragoza, Spain; 8 Unidad de Investigación, Centro de Salud La Alamedilla, Avda. Comuneros 27-31, 37003 Salamanca, Spain

**Keywords:** Actinidia, Fibrinogen, Cholesterol, HDL, Insulin resistance

## Abstract

**Background and aims:**

Among fruits, kiwi is one of the richest in vitamins and polyphenols and has strong anti-oxidant effects. We aimed to analyze the relationship between the consumption of kiwi and plasma lipid values, fibrinogen, and insulin resistance in adults within the context of a normal diet and physical-activity.

**Methods:**

Cross-sectional study. Participants (*N* = 1469), who were free of cardiovascular diseases, completed a visit, which included the collection of information concerning the participant’s usual diet and kiwi consumption using a previously validated, semi-quantitative, 137-item food-frequency-questionnaire. Fasting laboratory determinations included plasma lipids, fibrinogen and insulin resistance. Regular physical-activity was determined using accelerometry.

**Results:**

Consumers of at least 1 kiwi/week presented higher plasma values of HDL-cholesterol (mean difference 4.50 [95 % CI: 2.63 to 6.36]) and lower triglyceride values (mean difference −20.03 [95 % CI: −6.77 to −33.29]), fibrinogen values (mean difference −13.22 [95 % CI: −2.18 to −24.26]) and HOMAir values (mean difference −0.30 [95 % CI: −0.09 to −0.50]) (*p* < 0.05, for all comparisons) than those who consumed less than 1 kiwi per week. In an adjusted logistic regression analysis, this group had a lower odds-ratio for presenting plasmatic fibrinogen concentrations above 400 mg/dL (OR = 0.68, 95 % CI 0.49 to 0.95), HDL-Cholesterol plasma values below 45 mg/dL (OR = 0.57, 95 % CI 0.36 to 0.91) and a HOMAir above 3 (OR = 0.61, 95 % CI 0.37 to 1.00).

**Conclusions:**

Consumption of at least one kiwi/week is associated with lower plasma concentrations of fibrinogen and improved plasma lipid profile in the context of a normal diet and regular exercise.

## Background

A recent systematic review suggests that the advice to increase fruit and vegetable intake as a single intervention has favorable effects on cardiovascular disease (CVD) risk factors [1]. Recommendations to increase the consumption of fruit are based primarily on studies that indicate that fruit intake may reduce CVD risk through the beneficial combination of micronutrients, polyphenols and fiber [2–4], many of which have antioxidant and anti-thrombotic properties [5].

Among fruits, kiwi is one of the richest in vitamins and polyphenols and has strong anti-oxidant effects [6–8]. Phytochemical constituents of kiwi has health benefits including preventive effects against cancer cell growth [9, 10], oxidative DNA damage [11–13] in addition to cardiovascular protective properties [2, 14] and anti-inflammatory properties [12, 15]. The *in vitro* anti-oxidant effects of kiwi have been reported [6], and the consumption of kiwi exerts effects on platelets and plasma lipids, with the potential to increase the effectiveness of thrombosis prophylaxis [16] and improve the treatment of insulin resistance and diabetes [17].

Increasing evidence from epidemiological studies suggests that elevated plasma fibrinogen concentrations are associated with an increased risk of cardiovascular disorders, including ischemic heart disease, stroke and other thromboembolic events [18]. Similarly, high-density lipoprotein (HDL) cholesterol concentrations are a strong inverse predictor of cardiovascular events [19] and elevated blood triglyceride (TG) concentrations and insulin resistance predict CVD and all-cause mortality in the general population [20, 21].

Despite all of these findings, we found no studies that evaluated the association between kiwi consumption and plasma lipid and fibrinogen values or markers of insulin resistance in a large and heterogeneous population within the context of a regular diet and taking into account factors such as regular physical activity, alcohol consumption, body mass index (BMI) and the presence of lipid-lowering drugs.

The aim of this paper is to analyze the relationship between the consumption of kiwi and plasma lipid values, fibrinogen, and HOMAir in a large and heterogeneous sample of adults in the context of a usual diet and regular physical activity and taking into account the consumption of alcohol and other confounding factors.

## Methods

### Study design and population

A cross-sectional study was conducted. The findings presented in this manuscript represent a subanalysis of the EVIDENT study, whose purpose was to analyze the relationship between lifestyle and arterial aging in a cohort of healthy patients. The principal results were recently published elsewhere [22, 23], and the protocol (NCT01083082) can be found in a previously published study [24]. For the sample population, 1553 subjects aged 20 to 80 years (both sexes) were selected via random sampling from the patients of general practitioners at 6 health centers in Spain distributed throughout the geography of Spain. The exclusion criteria for the study included the following factors: known coronary or cerebrovascular atherosclerotic disease; heart failure; moderate or severe chronic obstructive pulmonary disease; musculoskeletal disease that limited walking; advanced respiratory, renal, or hepatic disease; severe mental disease; treated oncological disease diagnosed in the 5 years prior to the beginning of the study; terminal illness; and pregnancy. More details about inclusion/exclusion criteria are showed in the study protocol [24]. The recruitment and data collection period for the study was June 2010 to June 2012. For this purpose, we selected the 1469 individuals for whom kiwi consumption data was collected. A sample-size of 1469 patients was a sufficient sample for recognizing as statistically significant a difference in fibrinogen greater than or equal to 15 mg/dL between the two groups, assuming an alpha risk of 0.05 and a beta risk of 0.2 in a two-sided test. These groups were divided according to their consumption of kiwi (i.e., greater than or equal to 1 unit/week or less than 1 unit/week) with a ratio 1:3. According to this calculation, 353 subjects are necessary in the first group and 1059 subjects are necessary in the second group. The common standard deviation is assumed to be 87 mg/dL. The study was approved by the independent ethics committee of the Health Area of Salamanca, and all participants gave written informed consent for the study, according to the general recommendations of the Declaration of Helsinki.

### Variables and measurement instruments

#### Laboratory determinations

Venous blood sampling was performed between 08:00 and 09:00 a.m., after the individuals fasted and abstained from smoking and the consumption of alcohol and caffeinated beverages for the previous 12 h. The blood samples were collected in the respective health centers and were analyzed at the hospital of the city participating in the external quality assurance programs of the Spanish Society of Clinical Chemistry and Molecular Pathology. Serum total cholesterol, HDL-cholesterol and triglyceride concentrations were measured using standard automated enzymatic methods [25]. LDL cholesterol was estimated using the Friedewald equation when the direct parameter was not available. Fibrinogen concentrations were determined using an immunoturbidimetric assay [26]. The insulin sensitivity was determined using the Homeostasis Model Assessment Insulin Resistance (HOMAir) [27] in combination with the following formula: fasting glucose (mmol/l) X fasting insulin (mU/ml)/22.5.

#### Assessment of the normal diet and the consumption of kiwi

The regular consumption of kiwi, fruits and total calories in each participant’s diet was calculated from data obtained using a previously validated, semi-quantitative, 137-item food frequency questionnaire (FFQ) that was collected at the time of the interview [28]. The FFQ included 137 food items, and the participants indicated the frequencies of consuming the food items using an incremental scale with nine levels (i.e., never or almost never, 1–3 times per month, once per week, 2–4 times per week, 5–6 times per week, once per day, 2–3 times per day, 4–6 times per day, and more than 6 times per day). The reported frequencies of food consumption were converted to the number of daily intakes and multiplied by the weight of the portion size indicated. Kiwi consumption and other fruits are expressed in g/week. For statistical analysis, the sample was divided into two groups according to the weekly consumption of kiwi (i.e., greater than or equal to 1 unit/week or less than 1 unit/week).

#### Physical activity

Physical activity was estimated using accelerometry. The subjects wore Actigraph GT3X accelerometers (Actigraph, Shalimar, FL, USA) that had been validated previously [29]. The accelerometers were fastened with an elastic strap to the right side of the waist for 7 consecutive days, except while bathing and performing activities in the water. The data were recorded at 1-min intervals. Total physical activity was expressed in counts/min. The intensity of the physical activity (i.e., low, moderate, or high) was determined according to the cutoff points proposed by Freedson et al.[30].

#### Anthropometric measurements

Body weight was determined on two occasions using a Seca770 homologated electronic scale (Medical scale and measurement systems, Birmingham, United Kingdom) after calibration, with the patient wearing light clothing and no shoes. These readings were rounded to 100 g. Height was measured using a Seca 222 portable system (Medical scale and measurement systems, Birmingham, United Kingdom), which recorded the average of two readings; each patient was shoeless in the standing position. The values were rounded to the closest centimeter. Body mass index (BMI) was calculated as weight (kg) divided by height squared (m^2^).

#### Other measurements

A detailed description of the process by which the clinical data were collected was published previously [24], including an assessment of lifestyle habits, such as alcohol consumption and smoking.

According to the American Association of Clinical Endocrinologists’ guidelines for the management of dyslipidemia and the prevention of atherosclerosis, dyslipidemia was defined as the presence of total cholesterol ≥240 mg/dL or triglycerides ≥200 mg/dl or the use of lipid-lowering drugs [31].

### Statistical analysis

Continuous variables were expressed as the mean ± standard deviation, and categorical data were expressed as the frequency distribution. The *χ*2 test was used to compare categorical data. The difference in the means between 2-category of quantitative variables was analyzed using the independent samples Student’s *t*-test and Analysis of variance (ANOVA) for more two categories. The Bonferroni test was used for comparisons between subsequent pairs in the case of variables where the ANOVA showed a statistically significant difference. We performed two different analyses: an ANCOVA and a logistic regression model. In both analyses, we considered the following parameters as dependent variables: fibrinogen, HDL-Cholesterol, triglycerides and HOMAir; the consumption of at least 1 kiwi per week was considered as the independent variable. All models were adjusted for potential confounders, and the variables exhibited statistically significant differences between the two kiwi consumption categories (i.e., age, gender, total energy intake-Kcal, total fruit consumption, BMI, alcohol consumption and physical activity). Plasma lipids were also adjusted for the presence of lipid-lowering drugs. The data were analyzed using the Statistical Package for the Social Sciences software (SPSS), version 20.0 (SPSS, Chicago, IL, USA). A value of *P* < 0.05 was considered statistically significant.

## Results

Of the 1469 individuals (892 women, 577 men) included in this work, 355 (24.2 %) had a mean kiwi consumption of 1 kiwi or more per week (30.8 % of women and 13.9 % of men).

The mean age of the sample was 54.9 ± 13.8 years, with no differences in terms of kiwi consumption. With respect to the dependent variables of this study, the consumers of at least 1 kiwi per week presented higher plasma values of HDL-cholesterol (mean difference 4.50 [95 % CI: 2.63 to 6.36]) and lower triglyceride values (mean difference −20.03 [95 % CI: −6.77 to −33.29]), fibrinogen values (mean difference −13.22 [95 % CI: −2.18 to −24.26]) and HOMAir values (mean difference −0.30 [95 % CI: −0.09 to −0.50]) (*p* < 0.05 for all comparisons) than the subjects who consumed less than 1 kiwi per week. No differences in total cholesterol, LDL cholesterol and the percentage of individuals with diabetes or lipid-lowering treatment were observed (Table 1).

**Table 1:** Clinical characteristics and laboratory determinations of the study population

	Less than 1 kiwi/week		More or equal than 1 kiwi/week		
	*N* = 1114 (75.2 %)		*N* = 355 (24.2 %)		
	Mean or Number (*N*)	SD or %	Mean or Number (*N*)	SD or %	*p* value
Age (years)	54.7	14.0	55.7	12.6	0.203
Gender, male (*n*, %)	497	44.6	80	22.5	<0.01
Total cholesterol (mg/dL)	213.1	39.8	214.9	35.1	0.414
HDL-Cholesterol (mg/dL)	57.8	15.6	62.3	14.3	<0.01
LDL-Cholesterol (mg/dL)	133.0	36.4	132.7	31.9	0.894
Triglycerides (mg/dL)	125.7	118.9	105.7	75.5	<0.01
Fibrinogen (mg/dL)	376.3	89.9	363.0	82.0	0.019
HOMAir	1.97	1.69	1.67	1.19	<0.01
Diabetes (*n*, %)	88	7.9	28	7.9	0.992
Lipid-lowering drugs (*n*, %)	213	19.1	68	19.2	0.988

Among the modifiable lifestyle-related parameters, the consumers of at least 1 kiwi per week have a higher energy intake (mean difference 153.81 [95 % CI: 53.46, 254.17]) and a higher total fruit consumption (mean difference 211.53 [95 % CI: 183.93, 239.12]) (*p* < 0.05 both) than those who consume less than 1 kiwi per week. This group of individuals has a lower number of smokers (14.1 %), a lower alcohol intake (27.1 g/week) and lower mean BMI values (26.4 Kg/m^2^). In contrast, these individuals undergo more physical activity (268.3 counts/min) (Table 2).

**Table 2:** Total energy, fruit consumption and variables related to lifestyles in individuals according to their weekly consumption of kiwi

	Less than 1 kiwi/week		More or equal than 1 kiwi/week		
	*N* = 1114 (75.2 %)		*N* = 355 (24.2 %)		
	Mean	SD	Mean	SD	*p* value
Total energy (Kcal/day)	2405.5	857.6	2559.3	779.6	0.003
Total fruits (gr/week)	326.8	223.0	538.3	253.8	<0.01
Kiwi (g/week)	32.7	39.3	532.9	365.4	<0.01
Kiwi (g/week/weight)	0.47	0.59	7.90	5.49	<0.01
Vitamin C (mg/day)	191.0	105.6	284.9	113.5	<0.01
Smoking status (*n*, %)					
Smoker	267	24.0	50	14.1	<0.01
Exsmoker	343	30.8	103	29.0	
Non smoker	50.3	45.2	202	56.9	
Alcohol consumption (gr/week)	48.0	83.5	27.1	51.4	<0.01
BMI (Kg/m^2^)	27.2	4.6	26.4	4.4	0.005
Physical activity (counts/min)	246.4	137.1	268.3	121.1	0.011

*BMI* Body mass index

**Fig. 1:**
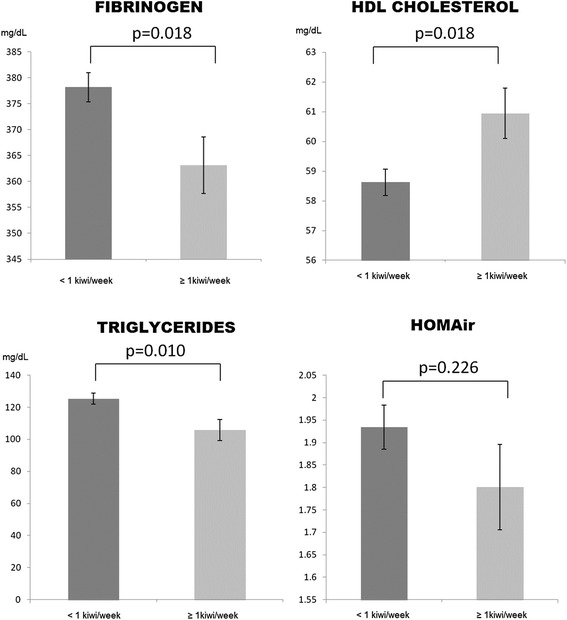
Fibrinogen, HDL-cholesterol, triglycerides and HOMAir according to kiwi consumption group. The bar graphs represent the marginal means and standard deviations adjusted for age; gender; physical activity (counts/min); total energy intake (kcal); total fruit consumption (gr/week), alcohol consumption (gr/week), body mass index and the presence of lipid-lowering drugs

In the ANCOVA analysis that was adjusted for confounders, the consumers of at least 1 kiwi per week had lower mean values of fibrinogen (363.1 ± 5.4 vs.378.2 ± 2.9 mg/dL, *p* = 0.018) and triglycerides (105.7 ± 6.5 vs. 125.4 ± 3.5 mg/dL, *p* = 0.010) than those who consumed less than 1 kiwi per week. In contrast, the HDL-cholesterol values of this group are higher than those of the group that did not consume kiwi (61.0 ± 0.8 vs. 58.6 ± 0.4 mg/dL, *p* = 0.018). The observed differences in HOMAir failed to reach statistical significance (1.93 ± 0.05 vs. 1.80 ± 0.09) (Fig. 1).

In the adjusted logistic regression analysis (Table 3), the consumers of at least 1 kiwi per week had a lower odds ratio for presenting plasmatic fibrinogen concentrations above 400 mg/dL (OR = 0.68, 95 % CI 0.49 to 0.95), HDL-Cholesterol plasma values below 45 mg/dL (OR = 0.57, 95 % CI 0.36 to 0.91) and a HOMAir above 3 (OR = 0.61, 95 % CI 0.37 to 1.00) than those who consumed less than 1 kiwi per week. The consumption of at least one kiwi per week was not significantly associated with the odds of having high triglyceride plasma concentrations.

**Table 3:** Logistic regression analysis considering fibrinogen, HDL cholesterol, triglycerides and HOMAir as dependent variables and consumption of kiwis as the independent variables

Dependent Variable:	OR	95 % CI: OR	*p*
Fibrinogen^a^	0.682	0.490 to 0.950	0.024
HDL-Cholesterol^b^	0.572	0.359 to 0.913	0.019
Triglycerides^b^	0.675	0.375 to 1.217	0.192
HOMAir^c^	0.609	0.371 to 1.001	0.050

Dependent variable: Fibrinogen (<400 mg/dL or ≥ 400 mg/dL); HDL-cholesterol (<45 mg/dL or ≥ 45 mg/dL); Triglycerides (<200 mg/dL or ≥ 200 mg/dL); HOMAir (<3 or ≥ 3)

^a^Model adjusted for age, gender (male = 1; female = 0), Total energy intake (Kcal/day), Total fruits consumption (gr/week), Alcohol consumption (gr/week), body mass index and physical activity (counts/min)

^b^Model adjusted for age, gender (male = 1; female = 0), Total energy intake (Kcal/day), Total fruits consumption (gr/week), Alcohol consumption (gr/week), body mass index and physical activity (counts/min) and lipid-lowering drugs

^c^Model adjusted for age, gender (male = 1; female = 0), Total energy intake (Kcal/day), Total fruits consumption (gr/week), Alcohol consumption (gr/week), body mass index and physical activity (counts/min), lipid-lowering drugs and presence of diabetes

Additionally, we have divided the sample into three groups according to their regular consumption of kiwi (non consumers, occasional consumers (less than 2 kiwis per week) or frequent consumers (more or equal than 2 kiwis per week). The frequent consumers have the highest mean value of HDL-cholesterol and the lowest mean value of triglycerides, fibrinogen and HOMAir. After the adjustment for confounders, this trend continues at the limit of the statistical significance, except for the HOMAir (Table 4).

**Table 4:** Plasma lipids, fibrinogen and HOMAir according to the regular consumption of kiwi in three groups. Results expressed as mean and 95 % confidence interval

	No adjustment							Adjusted						
	Non consumption		Occasional consumption		Frequent consumption			Non consumption		Occasional consumption		Frequent consumption		
	*N* = 594 (40.4 %)		*N* = 520 (35.4 %)		*N* = 355 (24.2 %)			*N* = 594 (40.4 %)		*N* = 520 (35.4 %)		*N* = 355 (24.2 %)		
	Mean	95 % CI	Mean	95 % CI	Mean	95 % CI	*p* value	Marginal Mean	95 % CI	Marginal Mean	95 % CI	Marginal Mean	95 % CI	*p* value
HDL-Cholesterol (mg/dL) ^ac^	57.1	55.8–58.5	58.5	57.2–59.9	62.3	60.8–63.8	<0.001	58.9	57.7–60.1	58.4	57.1–59.6	60.9	59.2–62.5	0.060
Triglycerides (mg/dL)^a^	129.2	119.5–139.0	121.7	111.3–132.1	105.7	97.8–113.6	0.007	125.2	115.5–134.8	125.0	115.2–134.9	106.5	93.6–119.5	0.050
Fibrinogen (mg/dL)	375.7	367.8–383.6	376.8	369.2–384.5	363.0	354.0–372.0	0.063	377.8	369.9–385.7	378.5	370.5–386.4	363.2	352.5–373.9	0.050
HOMAir^ab^	2.09	1.93–2.25	1.84	1.70–1.98	1.67	1.54–1.81	0.001	1.94	1.81–2.09	1.91	1.77–2.05	1.82	1.63–2.01	0.630

*p* values are for comparison of sub-groups by ANOVA test with Bonferroni’s post hoc comparisons. ^a^Difference between non consumers and frequent consumers; ^b^Difference between non consumers and occasional consumers; ^c^Difference between occasional consumers and frequent consumers

*p* values are for comparison of sub-groups by ANCOVA test adjusted by: age, gender, total energy intake-Kcal, total fruit consumption, BMI, alcohol consumption and physical activity

## Discussion

The consumption of at least one kiwi per week is associated with lower plasma concentrations of fibrinogen and triglycerides and higher HDL-cholesterol values in a large sample of adults. Similarly, the probability of having a HOMAir below 3 is lower among frequent users of kiwi. These results support the hypothesis that the particular characteristics of this fruit mediate anti-inflammatory and hypolipidemic effects and impact insulin resistance.

Fibrinogen plays a vital role in a number of pathophysiological processes in the body, including inflammation, atherogenesis and thrombogenesis [32]. A novel aspect of our results is the clear association between the intake of kiwi and plasma concentrations of fibrinogen. This relationship has not been described previously. Furthermore, this association is independent of the main determinants of fibrinogen concentrations (i.e., age, sex, obesity, an objective measurement of physical activity regularity, and alcohol consumption) [32] and remains significant after controlling for the total amount of calories and fruit per day. In addition, these results are only observed for kiwi consumption and not for the consumption of other fruits. The effects of kiwi consumption on platelet aggregation were studied previously by Duttaroy et al. [16] in a clinical trial that concluded that consuming two or three kiwi fruit per day for 28 days reduced the platelet aggregation response to collagen and ADP by 18 % compared to the controls (*P* < 0.05). Our results reinforce this relationship between the consumption of kiwi and platelet aggregation but take into account possible confounding factors that were not previously analyzed in a representative sample of the general population; this aspect was not considered in the study performed by Duttaroy et al. [16]. The proposed mechanisms of this effect include the infiltration of the vessel wall by fibrinogen, hemorheological effects caused by an increase in blood viscosity, increased platelet aggregation and thrombus formation. Furthermore, plasma fibrinogen is also a prominent acute-phase reactant. Elevated concentrations of fibrinogen, which may occur secondarily to inflammation and are implicated in cardiovascular risk, may operate in part by increasing the reactivity of platelets [33].

Kiwi consumption is related to higher plasma concentrations of HDL-cholesterol. This aspect is an important finding compared to the study of Duttaroy et al. [16] and reinforces the findings of Gammon et al. [15]. Moreover, this relationship is maintained in models after adjusting for important confounding factors, such as regular physical activity and the presence of lipid-lowering drugs. Further studies [34, 35] have analyzed the hypolipidemic role of kiwi extract in patients concluding that the consumption of two green kiwis a day had favourable effects on plasma HDL-Cholesterol. Both studies suggest that the most likely constituent to exert the observed effects are polyphenols and other components as vitamin C. Also, the green kiwi intervention significantly increased apoA1 (the main structural protein component of HDL) concentrations. The association with plasma triglyceride concentrations, which was reported by Duttaroy et al. [16] and was also found in our study, appears to be more uncertain. From a clinical viewpoint, after adjusting for confounding factors, the difference in mean plasma triglyceride values between the two kiwi consumption groups appears to be relevant. However, the differences in the frequency of consumption between the study performed by Duttaroy et al. and our study (i.e., 2–3 kiwis per day vs. at least 1 per week, respectively) may explain the different results obtained in the two studies. A regular diet typically includes a variety of fruits, including seasonal fruits. This variety in fruit consumption is the reason why we divided our study population into two major kiwi consumer groups: frequent consumers (i.e., at least 1 item per week) and occasional consumers or non consumers (i.e., less than once per week). The results of our study indicate an association of HDL-cholesterol and triglycerides with kiwi consumption within the context of a regular diet and in a large and heterogeneous sample, regardless of the intake of other fruits. However, like other studies, no association was observed with total cholesterol and LDL-cholesterol. This finding is consistent with other studies where none of the subjects who consumed kiwifruit exhibited a significant change in the mean of the serum LDL-Cholesterol compared with the control diet [34, 35]. Gammon et al. concluded that the difference in total fibre between the kiwi and control interventions may not have been sufficient to affect LDL-Cholesterol concentrations.

The acute effects of fresh fruit consumption on the values of insulin resistance has been examined previously [36]. However, the relationship between kiwi consumption in the context of a regular diet and insulin resistance, as measured by HOMAir, has not been previously evaluated. Abe et al. [17] tested in an “*in vivo*” experiment the effect of a kiwi methanol fraction on interleukin-6 and monocyte chemoattractant protein-1 mRNA levels. These molecules are proinflammatory adipocytokines that are induced by insulin resistance. The relationship between kiwi consumption with the insulin resistance measured by the HOMA index is less clear. Although there is a tendency after adjustment for confounding factors, showing lower HOMA index in those with frequent consumption of kiwi, this difference did not reach statistical significance. However, we found a relationship between insulin resistance and occasional and frequent consumption of kiwi when we established a cutoff point of three in the HOMAir. This cutoff was chosen because it is representative of the general population that visits health centers and because in a study of Spanish non-diabetic population, the diagnostic of insulin resistance was established considering a similar cutoff point [37].

The main limitation of this study is the cross-sectional design, which prevents the establishment of causal relationships between the consumption of kiwi and fibrinogen and plasma lipids. Second, the kiwi intake data was based primarily on a food frequency questionnaire, which was designed to assess the habitual diet by asking about the frequency of a limited number of food items. Finally, we controlled the effect of potential confounders on the results such as variables related to lifestyles, but other factors may affect the relationship between kiwi consumption and fibrinogen and plasma lipids.

## Conclusions

In conclusion, a frequent consumption of kiwi in the regular diet is associated with lower plasma concentrations of fibrinogen, an improved plasma lipid profile. Further interventional studies are needed to define the effects of kiwi consumption on insulin resistance and cardiovascular outcomes.

### Availability of supporting data

The data set supporting the results of this article are included within the article and its additional files.
